# Questionnaire-Based Assessment of Wild Boar/Domestic Pig Interactions and Implications for Disease Risk Management in Corsica

**DOI:** 10.3389/fvets.2017.00198

**Published:** 2017-12-01

**Authors:** Ferran Jori, Anne Relun, Bastien Trabucco, François Charrier, Oscar Maestrini, David Chavernac, Daniel Cornelis, François Casabianca, Eric Marcel Charles Etter

**Affiliations:** ^1^CIRAD, UMR Animal, Santé, Territoires, Risque et Ecosystèmes (ASTRE), Montpellier, France; ^2^ASTRE, Univ Montpellier, CIRAD, INRA, Montpellier, France; ^3^BIOEPAR, ONIRIS, INRA, Nantes, France; ^4^INRA, Laboratoire de Recherches sur le Développement de l’Elevage (LRDE), Corte, France; ^5^Epidemiology Section, Department of Production Animal Studies, Faculty of Veterinary Science, University of Pretoria, Pretoria, South Africa

**Keywords:** *Sus scrofa*, wild boar, pig farming, Corsica, disease management, contacts, transmission, human practices

## Abstract

Wild boars and domestic pigs belong to the same species (*Sus scrofa*). When sympatric populations of wild boars, feral pigs, and domestic pigs share the same environment, interactions between domestic and wild suids (IDWS) are suspected to facilitate the spread and maintenance of several pig pathogens which can impact on public health and pig production. However, information on the nature and factors facilitating those IDWS are rarely described in the literature. In order to understand the occurrence, nature, and the factors facilitating IDWS, a total of 85 semi-structured interviews were implemented face to face among 25 strict farmers, 20 strict hunters, and 40 hunting farmers in the main traditional pig-farming regions of Corsica, where IDWS are suspected to be common and widespread. Different forms of IDWS were described: those linked with sexual attraction of wild boars by domestic sows (including sexual interactions and fights between wild and domestic boars) were most frequently reported (by 61 and 44% of the respondents, respectively) in the autumn months and early winter. Foraging around common food or water was equally frequent (reported by 60% of the respondents) but spread all along the year except in winter. Spatially, IDWS were more frequent in higher altitude pastures were pig herds remain unattended during summer and autumn months with limited human presence. Abandonment of carcasses and carcass offal in the forest were equally frequent and efficient form of IDWS reported by 70% of the respondents. Certain traditional practices already implemented by hunters and farmers had the potential to mitigate IDWS in the local context. This study provided quantitative evidence of the nature of different IDWS in the context of extensive commercial outdoor pig farming in Corsica and identified their spatial and temporal trends. The identification of those trends is useful to target suitable times and locations to develop further ecological investigations of IDWS at a finer scale in order to better understand diseases transmission patterns between populations and promote adapted management strategies.

## Introduction

Outdoor pig farming is becoming a widespread activity in many European countries driven by public demand for more ethical and natural approaches to produce pigs and a higher quality of pork products. In the French island of Corsica, pig production of indigenous breeds raised in extensive pastures and forests (chestnut and oaks) for the processing of high quality dry cured meats is a traditional activity. The processed pork products obtained through this type of farming are highly ranked among tourists and French consumers. This type of production has important socio-economic, ecological, and cultural benefits and is being promoted to revert to traditional farming practices in Corsica.

The natural and vast pig farm environment is often shared with an abundant population of wild boars, feral pigs, and hybrids resulting of cross breeding between these different forms of *Sus scrofa* that cohabitate in the Corsican Mediterranean forests. Hunting is extremely popular in the island and practiced by approximately 17,000 licensed local hunters and 250 hunting teams who regulate wild boar populations with an estimated annual offtake of 30,000 (1, 2). Such socio-ecological context provides numerous opportunities for direct (synchronous) or indirect (asynchronous) interactions between wild/feral and domestic suids (IDWS), which are known to be responsible for the maintenance and transmission of many infectious diseases circulating between wild and domestic compartments such as Hepatitis E virus, bovine tuberculosis, or trichinellosis (3–5). Equally, Aujeszky disease, has been eradicated from domestic pigs in continental France, but remains present in Corsican herds as result of IDWS (6). Last but not least, IDWS are reported to contribute to the maintenance of African swine fever in the neighboring island of Sardinia, which has remained endemic for more than 35 years (7, 8). However, despite their suspected epidemiological importance, no studies have ever analyzed the characteristics, drivers, or patterns of occurrence of IDWS in the framework of a complex Mediterranean socio-ecosystem (9). Knowledge on the spatial and temporal patterns of IDWS can be used to target preventive disease management measures and to reduce the risk of transmission more efficiently at specific times and locations.

Several ecological or laboratory methods have been used to assess interactions between wild and domestic animals such as telemetry (10, 11), camera trapping (12), or biomarkers (13, 14). In addition, the collection of local knowledge by interviews with stakeholders has been reported as a useful and cost effective tool to collect qualitative and quantitative information on events that are otherwise difficult to observe (15–17). Questionnaires have proven useful to assess potential contacts between wildlife and livestock in different parts of the world (17–19). They have been successfully used to assess IDWS in Switzerland (20), and more recently in Uganda (21).

In this study, we implemented questionnaires in the Corsican socio-ecological context of outdoor pig farming with the specific aims of (a) describing the nature, frequency, duration, and seasonality of IDWS, (b) identifying their seasonal and spatial drivers, and (c) identifying potential hunting and farming practices that could facilitate or reduce their occurrence.

## Materials and Methods

### Study Area

Corsica is located off the western shores of the Italian peninsula, 11 km north of the Italian island of Sardinia. Its territory, divided in the two Corsican Provinces (North and South Corsica), is sparsely populated (32 inhabitants/km^2^) and its economy is mainly based on services closely linked with tourism (22). Pigs are mostly reared in mountainous and semi-mountainous areas, called “*Pieve*” (“diocese” in Corsican language) which include the 14 main pig production areas distributed within 6 major pastoral areas with an average size of 557 km^2^ (23). In the eastern part of the island, the valleys reach a plain (“*Plaine orientale*”) dominated by more intensive pig production. The highland habitats in the center form a single chain of 21 summits reaching more than 2,000 m (6,600 ft) above sea level (a.s.l.). The slope of the terrain varies significantly from area to area. In order to produce typical Corsican ham, pigs are left roaming in this mosaic of oak and chestnut forests in autumn, whereas in summer, pigs are traditionally kept in often unfenced grass pastures and beech forests found in altitude (24). The pasture vegetation reflects the influence of both Mediterranean and mountain climates, with shrubs (“*maquis*”), a mixture of rapidly growing evergreen herbs, bushes, and small trees, holm oak (*Quercus ilex*), cork oak (*Quercus suber*), and olive trees up to 600 m, chestnut trees between 600 and 1,000 m, and mostly grass and beeches above 1,000 m a.s.l.

### Study Design

**Figure 1:**
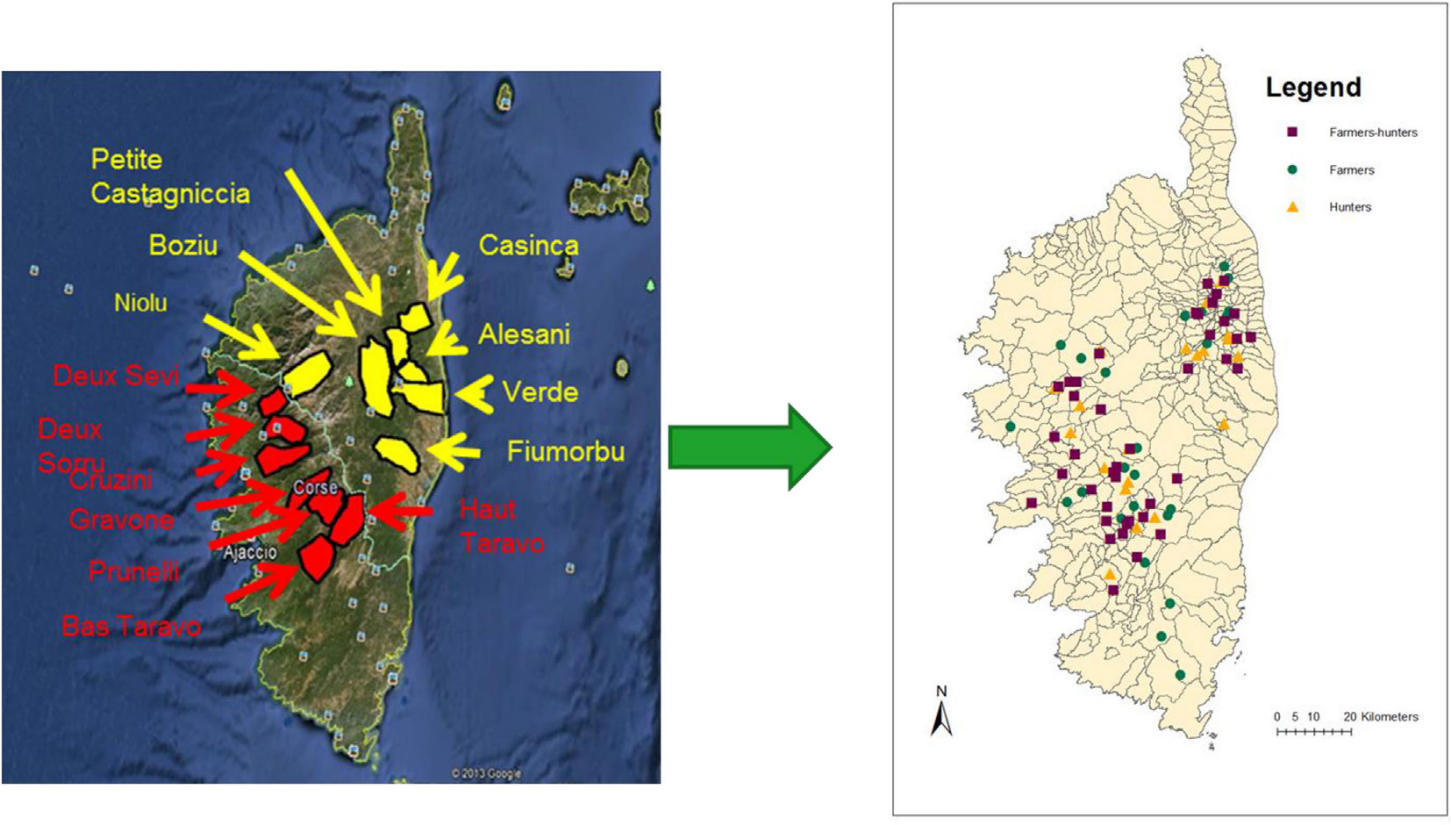
Two maps of Corsica showing the 14 main outdoor pig production areas in the island (left) and the spatial distribution of the interviewed stakeholders (right).

A cross-sectional study was conducted between March and October 2013 in the 14 main extensive pig production areas (Figure 1), which encompass 287 extensive farms registered in 50 Corsican municipalities. The sampling frames were based on two databases facilitated by (i) the Animal Health Associations (“*Groupements de Défense Sanitaire*”) and (ii) the Corsican Hunters Federation, respectively, and consisted of a list of all registered commercial outdoor pig farms (372 properties) and 17,000 licensed hunters.

Considering the time and resources available for the study, we estimated that 70 pig farmers and 20 hunters could be interviewed. The distribution of the farmers in the island was stratified according to the importance of 14 main extensive pig-farming areas within the two Corsican Provinces (55% in Southern Corsica and 45% in Northern Corsica). In each production area, extensive farmers were randomly chosen from the lists provided and contacted by phone to check if they were still active and would be willing to take part in a face-to-face interview on farming practices and IDWS. Farmers were classified as hunting farmers (HF), if they dedicated at least 2 days a week to hunting activities and had a hunting license or as strict farmers (SF) if they had little or no hunting activity. In addition, a sample of 20 hunters (not farmers) operating in the same municipalities as the farmers was randomly chosen from the Hunters Federation list in order to obtain responses from farmers and hunters in the same municipalities.

### Data Collection

The selected farmers and hunters were subjected to a semi-open face-to-face questionnaire interview. The questionnaire was designed to reveal stakeholders’ knowledge and perception of IDWS and to collect data on their herd management or hunting practices that could influence IDWS (copy of the questionnaire available).

All stakeholders were asked to recall their field observation of proximity of wild pigs to pig estates and of direct IDWS events, as well as their herd management or hunting practices within the previous 12 months.

For field observation of direct (i.e., synchronous) IDWS events, stakeholders were asked to recall specifically on mating activities between domestic and wild pigs (hereafter referred as sexual IDWS), fighting between domestic and wild pigs (agonistic IDWS), and foraging together around common food or water (trophic IDWS). They were asked to specify the duration of each events, their annual frequency and seasonality, and to give some additional information on the circumstances of such IDWSP (for example, did you observe mating between wild boar and a domestic sow or the inverse?).

To avoid confusion, we defined wild *Suidae* as the term encompassing any animal living in the wild without an owner, including pure wild boars, feral pigs, and hybrids. We defined a feral pig as a domestic pig that escaped its original farming premises, had no owner, and roamed free without confinement. Hybrids were distinguished from “pure wild boars” on the basis of presence of phenotypical or behavioral indicators such as patches of white or clear coat, the shape of the ears, or their ability in confronting hunting dogs (25).

Farmers were asked about the number of domestic boars injured, and the number of hybrid litters in the previous 12 months, as well as on their herd management practices, focusing on reproduction, feeding, confinement, and disposal of carcasses, offal, and animal waste. Maps and a 2-year round calendar were used to collect information on the location of pastures and neighboring farms and the production schedule for the whole year. Hunters were asked on their hunting practices, focusing on the type or intensity of hunting activity (number of hunting days per week, shooting feral pigs, or driven hunts).

The questionnaires were pre-tested with three pig farmers and three hunters and modified accordingly before being implemented in the field. The final version of the questionnaire was administered by two interviewers who were involved in its design. The interviewers took notes during the interview and the data were entered in a LimeSurvey database (26). Answers to open questions were coded according to the analysis methods used in human sciences (27).

### Ethics

To the best of our knowledge, the implementation of questionnaires among French citizens does not require a specific Ethics review process. Participation of hunters and farmers to the interviews was done on a voluntary basis after phone call contact. Participants were informed in advance about details of how the data would be used, assuring anonymity, and informed consent was obtained.

### Data Analysis

Data collected through the questionnaires consisted on a series of binary categorical or quantitative continuous variables describing the intensity and frequency of IDWS (non-specific, sexual, agonistic, and trophic) and a large number of human practices implemented by farmers (SF or HF), potentially influencing IDWS. For hunters, the number of practices was lower and linked to the type or intensity of hunting activity.

Four annual frequencies of IDWS (non-specific, sexual, agonistic, and trophic IDWS) and two quantitative variables (number of observations of wild boar near the farm and number of hybrid litters during the previous year) were used to assess the intensity of interactions per farm and compare those frequencies between stakeholder categories [strict hunters (SH), SF, or HF] during the previous 12 months to the interview. For hunters, we considered the same four annual frequencies of observation of IDWS, the rate number of hybrids/total number of suids shot, and the number of hunting days during the previous 12 months to the interview. Each indicator was scored as 1 (1–3 times), 2 (4–6 times), 3 (6–10 times), or 4 (>10 times), depending on the frequency of events reported by farmers and hunters during the previous 12 months. The sum of these four indicators was used to calculate an interaction index which was used to classify farms or hunting properties with a quantitative value ranging from 0 (lowest level of IDWS) to 16 (highest level of IDWS).

A preliminary selection of the variables linked to those practices was made by suppressing binary variables for which less than 5% of respondents gave a positive (or negative) answer or by suppression of variables for which at least 20% stakeholders did not provide any answer (28). Other variables from the questionnaire were removed due to lack of discrimination (all the same answer). After selection, analysis of farming practices was performed on 34 selected variables grouped in 6 sets of data and analysis of hunting activities on IDWS was performed on 10 variables from the hunter’s questionnaire (Table 1).

**Table 1:** Presentation of the 30 variables selected from farmers and 10 variables selected from hunters on which principal component analysis and multiple component analysis was performed.

Farmer characteristics	Pasture management	Reproduction management	Hunter characteristics and management	Carcass management	Interactions between wild suidae (IDWS)
Farming is the main activity (Y/N)	Maximum surface of the outdoor area (<50/>50 ha)	Births during all the year (Y/N)	Number of pig farms in the neighborhood (quantitative)^a^	Carcasses left outdoor (Y/N)	WB in proximity (seen in the farm/estate) (Y/N)

Pork produced as controlled designation of origin (Y/N)	Pastures are totally fenced (Y/N)	Mating in non-fenced areas (Y/N)	Practice of beat hunts (Y/N)^a^	Offal left outdoor (Y/N)	WB/pig interaction (Y/N)

Size (<100/>100 ha)	Share of pastures with other pig farmers (Y/N)^a^	Sterilization of sows (Y/N)	Number of hunting days/week (quantitative)^a^	Home slaughtering (Y/N)	IDWS frequency (seen more than 4 times a year) (Y/N)^a^

Breed “Nustrale” (Y/N)	Use of summer pastures (Y/N)^a^	Reproductive stock sold (Y/N)	Shooting feral pigs (Y/N)^a^		Sexual interactions (Y/N)

Cross bred with Corsican breeds (Y/N)	Additional food supply all year (condensed) (Y/N)	Fattening offspring sold (Y/N)	Ratio hunted hybrids/total hunted pigs^a^		Agonistic interaction (Y/N)

Farming experience (<20/>20 years)			Farmer (Y/N)^a^		Trophic interactions (Y/N)

Age (<40, >40 years)					Sexual interactions frequency (>4 times) (Y/N)

Isolation of the farm (nearest other pig farm ≥10 km)					Agonistic interaction frequency (>4 times) (Y/N)

Hunter (Y/N)					Trophic interaction frequency (>4 times) (Y/N)

					Presence of hybrids in the litters (Y/N)^a^

					Keep the hybrids born in farm (Y/N)

					Observation of wounds (>1) on boars (Y/N)

*^a^Variables used for the analysis of hunters practices in relation with IDWS*.

Quantitative values of frequency and seasonality of IDWS were calculated as medians and interquartile ranges (IQR), percentages and histograms of IDWS reported by month, and stakeholder categories. Potential differences in frequency, seasonality, or duration of reported IDWS by different observers (SF, SH, and HF) were assessed using the ANOVA test for inequality of means. Differences were considered significant when *p* values were lower than 0.05. Seasonal variations were detected by analyzing the frequency of observations across time in the questionnaire responses.

The geographical coordinates of the farming and hunting areas were not always available, but we could obtain the name of the municipality where each farmer and hunters was living. Therefore, we used this information as a proxy of farming area and hunting grounds. For mapping purposes, an average municipality IDWS index was calculated as the ratio between the sum of the interaction indexes per farm or hunting area/the number of interaction indexes observed in each municipality. Those IDWS municipality indexes were then manually classified with ARC-GIS, as high (10–6), medium (45–3), low (1–2), and null (0) in order to obtain equally represented categories (null 20%, low 22%, medium 34%, and high 25%). Similarly, assessed municipalities were equally classified by the average altitude in meters above sea level (m) in four categories ranging between <100, 100–350, 350–700, and >700 m. The association between those categories (IDWSP intensity vs municipality altitude) was measured using a simple Chi square test.

Multivariate exploratory data analysis including multiple factorial analysis (MFA) and principal component analysis (PCA) were conducted to identify farming and hunting practices correlated with each other or with the intensity of IDWSP variables (outcome). Principal components (or dimensions) were produced based on the linear combination of the variables. The selection of the dimensions was based on a combination of the Kaiser–Guttman criterion (eigenvalues >1) and the screen test (29, 30). The relative importance of each component is expressed by the variance (i.e., eigenvalue) of its projection or by the proportion of the total variance expressed. Variables or individuals are projected on planes which axes are represented by these principal components. Close proximity of the variables or individuals on one plane of the projections suggests that the variables/individuals are positively correlated while position on opposite quadrant of the plane suggests negative correlation. At individual level, these analyses allow to assess the variability or similarity between individuals considering simultaneously all their variables and to look for potential clusters (31). Variables used in the PCA were created using either categorical variables (but reducing the categories to a maximum of three) or quantitative variables categorized using mode or thirtiles of their distribution. Two or three categories were used in order to avoid variables to overweight each other in the analysis (32, 33). Two out of the 10 variables kept for the hunter’s data analysis were considered as supplementary variables. These two variables were not used to build the principal components but were added on the correlation circle afterward. Considering that some variables of the farmer’s analysis could be organized into groups, MFA was preferred to PCA. MFA could be considered as a weighted PCA where the influence of each group of variables was balanced in the analysis.

To quantify the level of correlation between some variables relative to farming or hunting practices and some indicators of interaction identified in the PCA, we extracted the correlation matrix from the PCA. The correlation between two variables was expressed by a correlation coefficient (*r*) and was considered significant if the *p* value was lower than 0.05. The use of correlation coefficient even for categorical variables with two or more modalities is possibly due to the statistical relationship between correlation coefficient and the Chi^2^ (33). Descriptive uni- and multivariate analysis were performed with R software version 2.15.3 using the package FactoMineR for explanatory data analysis (34).

## Results

A total of 85 persons from 56 different municipalities were interviewed (Figure 1). Among those, 29.5% (*n* = 25) were SF, 23.5% (*n* = 20) were SH, and 47% (*n* = 40) were at the same time farmers and hunters (HF).

### Pig Management Practices

Pig management practices from Corsican traditional farmers have been recently described in detail (35). “Nustrale” pigs, the selected and recognized local breed which is subject to selection programs were the most common breed, followed by the “Corsican-type” pigs, which refers to the original local non-selected pig population and cross-bred animals from different origins. Median time of experience for farmers was 25 years IQR [15–30]. The majority were farrow-to-finishers. The median herd size was 115 pigs [89–159] encompassing 10 adult sows [8–20], 2 boars [1–3], and 100 fattening pigs [89–159]. There was no significant difference between North and South Corsica with respect to herd size among SF or HF.

In relation to herd management, 100% (*n* = 65) of farmers (SF + HF) kept their pigs outdoors in areas with a median surface of 60 ha [20–700]. Producers mixed their herds in grazing areas in 49% cases (*n* = 32) and only 23% of farms were totally fenced (*n* = 15). The type of fence used by 86% of them (13/15) was a simple fence, while one farmer used a double fence and another one an electric fence. Among the 65 interviewed farmers, 17% moved pigs to high pastures in summer and left them free ranging in altitude for several weeks (*n* = 11). A total of 52% provided supplementary feed all year round (*n* = 34), 45% of them (*n* = 29) only in summer and three of them did not supplement food at all.

Around 15% of the farmers interviewed (10/65) reported that mating of sows occurred in free ranging natural conditions, while the others (85%) kept their reproductive females confined in outdoor specific paddocks for monitored reproduction. Most farrowing occurred from April to August. About half the farmers (47%) said that their sows farrowed once a year, mostly in spring and summer (39%) and a minority (8%) in autumn and winter. A similar proportion (42%) of farmers synchronized mating time so that farrowing could occur twice a year, once in the spring–summer season and once in the autumn–winter season. In a limited number of farms (11%; 7/65), farrowing was spread over the year. All farmers castrated their fattening males at weaning age (3 months). Of all farmers, 34% (22/65) also spayed reproductive sows of different ages (4–48 weeks) aimed for fattening, to avoid undesired mating with domestic or wild boars. Furthermore, 43% (28/65) of the farmers (including SF and HF), dumped carcass offal of domestic pigs in the environment. This was reported to occur mostly during the pig-butchery period (between November and April).

In addition, a non-negligible proportion of farmers slaughtered their animals at home (17%, 11/65) and not at the slaughterhouse (83%). Among those, 81% of them reported leaving carcass offal in nature without disposal. A limited number of farmers (8%, 5/65) even reported feeding carcass left overs directly to their pigs. Regarding management of dead animals, 47% (38/65) of the farmers transported the carcasses to a specific area for disposal, 32% (21/65) reported leaving dead carcasses outdoors without any management, while 13% (6/65) of the farmers did not answer this question.

Thirteen farmers (12 HF and 1 SF) reported the deliberate use of supplementary entire males around the sows on pasture, as a specific strategy to mitigate sexual interaction with wild boar (males are supposed to cover the sows and hence to avoid the heats attracting wild boars).

### Hunting Practices

The following results were extracted from the analysis of questionnaires including 20 SH and 40 HF that provided quantitative data on the IDWS. The median age of a hunter in our study was 53 [46–58] years. The median number of days per week dedicated to hunting during the hunting season (beginning of September to the end of February) was 2 [2–3]. The most widespread hunting method was the driven hunt, practiced by 88% of hunters. A hunting team was reported to encompass a median of 10 persons [8–15] and 15 dogs [8–20]. Hunting alone and/or from a hide were practiced by 30 and 16% of the hunters, respectively. The median number of wild pigs shot per hunting team per year was 100 individuals [65–137] encompassing 53% of suspected hybrid pigs and 47% of suspected pure wild boars. Feral domestic pigs were not a preferred target, being only shot occasionally by 25% of the hunters to control their population at a median range of 1–2 individuals per year. A large majority of the SH (85%) declared dumping hunted pig offal in the natural environment during the hunting season.

### Types and Occurrence of IDWS

**Figure 2:**
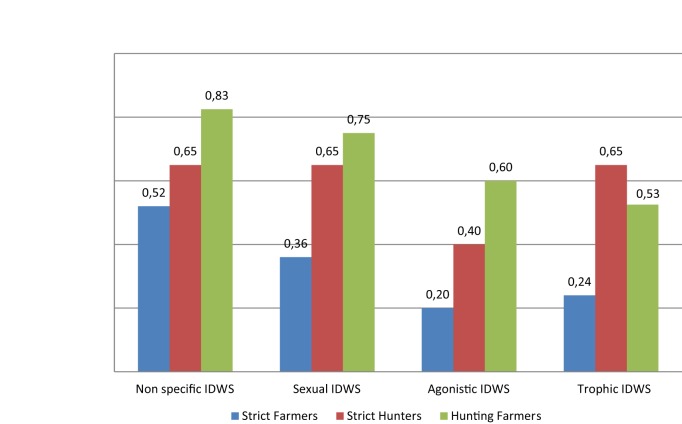
Proportion of responses given by the different categories of stakeholder interviewed (SH, SF, and HF), regarding the different kinds of interaction observed. SF, strict farmers; SH, strict hunters; HF, hunting farmers.

The proportions of the different types of IDWS reported by each stakeholder category are presented in Figure 2. All the farmers reported having wild boars close (less than 500 m) from their farm area. More than 3/4 (51/65) of the farmers (including SF and HF) and a majority of hunters (SH) (18/20) reported having seen wild boars within the premises of pig farms. Non-specific IDWSs were observed by 69% of the persons interviewed (52% of SF, 65% of SH, and 83% of HF). More than 2/3 of the interviewed individuals had observed some kind of IDWS (1–3 times per year in 40% of the cases, 4–6 times per year in 10% of the cases, and more than 7 times per year in 12% of the cases). Specific IDWS most commonly reported were sexual (61%, 52/85 interviewees), followed by trophic (47%, 40/85) and agonistic (43%, 37/85) IDWS.

### Sexual Interactions (Mating)

The observation of sexual IDWS were reported by 61% (52/85) of the respondents, encompassing 36% of SF, 65% of SH, and 75% of HF. In median, stakeholders observed sexual interactions 5 times [2–8] per year and differences between stakeholder categories were not significant. The median reported duration of courtship was 2.3 days [2–2.5] and the perceived duration was significantly higher among SH (*p* < 0.02). The median duration of a sexual intercourse observed was 5 [5–15] min and differences between stakeholders were not significant. Sexual IDWS were reported to occur when a domestic sow was mated by a wild boar or feral pig in the farm paddocks (63%) or in unfenced areas (32%), while wild or feral sows being sexually harassed by domestic boars in farm paddocks or outside was only reported by 9 and 1% of the farmers, respectively. More than half of the farmers interviewed (57%, 37/65) reported births of hybrid litters in their premises because of those sexual contacts. Those included eight farmers who had not reported the observation of sexual interactions, so the number of farmers reporting some kind of sexual IDWS (mating or hybrid litters) was 70% (60/85). The median number of hybrid litters reported/year in those 37 farms was 2 [1–4]. Among the 37 farmers who reported hybrid litters, 21 (57%) did not keep those hybrids, while 43% kept them for consumption (7/16) or sold them in the market (9/16).

### Agonistic Interactions (Fighting)

The observation of fights between wild and domestic boars when trying to mate with domestic sows on heat was reported by 44% (37/85) of the respondents, encompassing 20% of SF and 60% HF and 40% of SH. In addition, there were five farmers (3HF + 2SF) who reported wounds in domestic boars because of those fights, so the total number of respondents having observed some kind of agonistic interaction was 49% (42/85). The median reported frequency was 2.5 times/year [2–4] and the median reported duration of those boar fights was 1 h [0.4–2]. In both cases, measures provided by different stakeholders were not significantly different (Table 2). In 84% of the observed fights, wild boar succeeded in chasing their domestic opponents away. The occurrence of wounds resulting from bites during fighting was reported by 62% (23/37) of the farmers who observed fighting interactions. The median annual frequency of observation of boar wounds was 2.5 [2–4] per farm.

**Table 2:** Frequency and duration of different types of interactions between wild or feral pigs and domestic pigs reported by different stakeholders in Corsica in 2013 [SF, strict farmers; SH, strict hunters; HF, hunting farmers (i.e., the farmers hunting at least 2 days a week)].

Type of interaction observed	SF (*n* = 25)	SH (*n* = 20)	HF (*n* = 40)	Overall (*n* = 65)
**Sexual**				
Number of observers (%)	9 (36)	13 (65)	30 (75)	52 (80)
Median annual frequency (IQR)	2.3 (1–7)	5 (2–6)	5.5 (3–10)	5 (2–8)
Median duration of courtship (days) (IQR)	2 (2–2.5)	2.5 (2.5–3)*	2.3 (2–2.5)	2.5 (2.3–3.3)
Median duration of intercourse (min) (IQR)	5 (5–15)	10 (5–15)	5 (5–15)	5 (5–15)

**Agonistic**				
Number of observers (%)	5 (20)	8 (40)	24 (60)	37 (57)
Median annual frequency (IQR)	3.5 (2–4)	2 (2–3)	3 (2–5.5)	2.5 (2–4)
Median duration of fights (h) (IQR)	1 (0.5–2)	0.5 (0.4–2.5)	1 (0.2–1.5)	1 (0.4–2)

**Trophic**				
Number of observers (%)	6 (24)	13 (65)	21 (53)	40 (62)
Median annual frequency (IQR)	2 (1–3)	3 (2–10)	3 (1–3)	3 (1–3)
Median duration of foraging together (days) (IQR)	20 (7–120)	5 (1–19)	19 (2–75)	7 (2–75)

*Indicates significant differences (*p* < 0.05).

*IQR, interquartile ranges*.

### Trophic Interactions (Foraging)

Wild and domestic suidae were reported to share foraging sites by 47% (40/85) of the persons interviewed, including 24% of SF, 53% of HF, and 65% of SH. This was reported to be observed three times per year [1–3] and the interaction could last for a median of 7 days [2–75].

### Seasonality of IDWS

**Figure 3:**
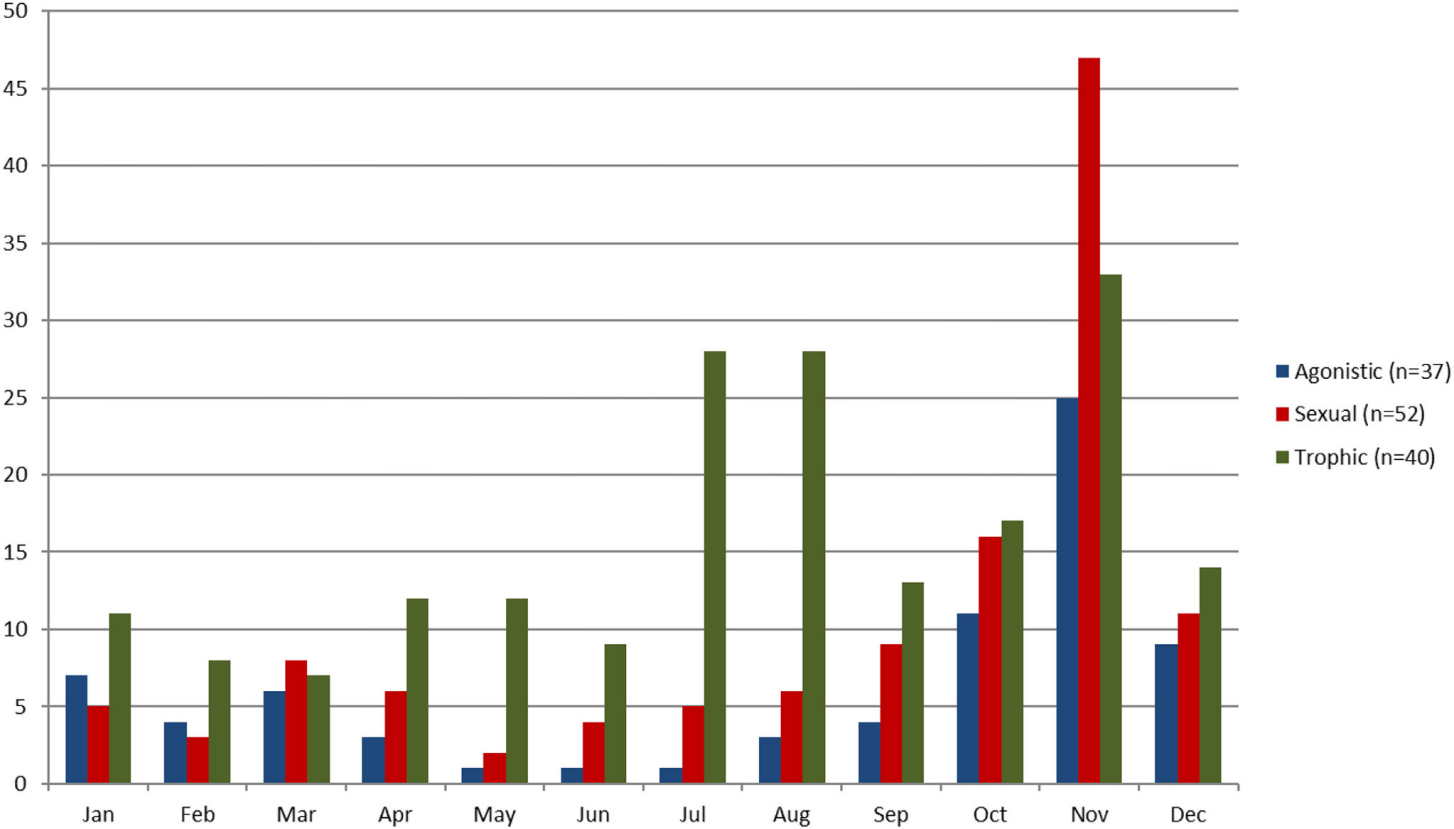
Histogram of the seasonality of IDWS (sexual, agonistic and trophic) in Cosrsica during 2013 observed by farmers and hunters.

In terms of seasonality, most of the interactions were observed in the autumn months, with a peak of observation in November. Observations from SH coincided with the autumn months while SF mostly reported interactions on other seasons (Figure 3).

Sexual IDWS were observed mostly (70%) in autumn (September to December) and 30% in winter (January to March). Fights were also reported to occur in autumn, and mostly in November coinciding with the period of estrus of the sows. Trophic interaction was reported to occur at different periods of the year depending on the availability of different berries and fruits (Figure 3). Most interactions were reported to occur from October to April around fallen oak fruits (45/55, 81%), from October to January around the chestnuts (47/61, 77%), from May to August around summer berries (39%, 13/33), and from August to October around beech nuts (8/30, 26%).

### Spatial Distribution of IDWS

**Figure 4:**
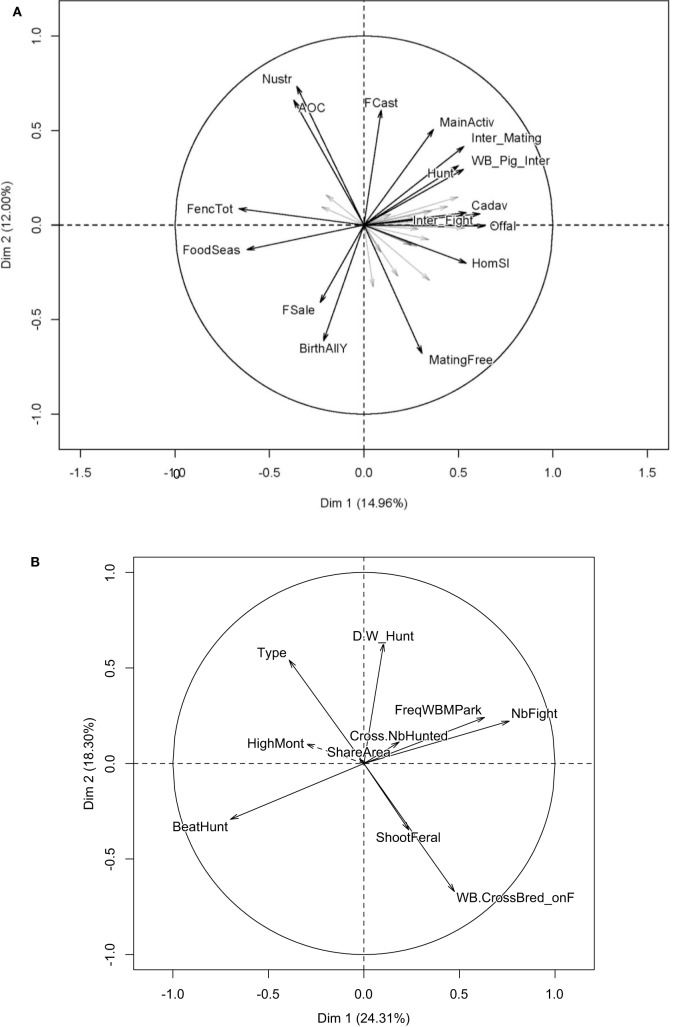
Potential associations between the different practices according to the multivariate exploratory data analysis: **(A)** correlation circle of the MFA for farming practices (including SF ad HF) and **(B)** correlation circle of the PCA for hunters (including SH and HF). Legend for the variables in **(A)** AOC: farmers involved in quality recognition process; BirthAllY: no reproductive synchronization; FCast: sterilization of females; FencTot: total fencing of farm perimeter; FoodSeas: additional food supply; FSale: sale of females; HomSl: slaughtering of pig on the farm; Hunt: farmers practicing hunting activity; Cadav: disposal of the carcasses outdoor (no specific area); Inter_Fight: observation of fighting interaction (Y/N); Inter_Mating: observation of mating interaction (Y/N); MainActiv: farmers having pig farming as main activity; MatingFree: mating of sow in non-fenced area; Nustr: farmers having only Nustrale breed in the farm; Offal: disposal of carcass left-over outdoor (no specific area); WB_Pig_Inter: observation of any IDWS (Y/N). Legend for the variables in **(B)** BeatHunt: practice driven hunt (Y/N); rossNbHunted: ratio of cross-bred/wild boar by hunt; D.W_Hunt: number of hunting days per week; FreqWBMPark: annual frequency of observed sexual interactions. HighMont: farming in mountainous areas; NbFight: annual number of fights between wild and domestic boars; ShareArea: share of pastures with other pig farmers; ShootFeral: hunter shooting feral pigs (Y/N); Type: pure hunter (no farming); WB.CrossBred_onF: observed presence of wild pigs around the farm. SF, strict farmers; HF, hunting farmers; SH, strict hunters; MFA, multiple factorial analysis.

Figure 4 shows the map with the classified IDWS indexes per municipality. A majority (59%, 33/56) of the municipalities assessed reported medium to high level of IDWS. There was a trend of lower interactions toward the coastal areas and farms located at lower altitude (Bas Taravo and lower areas of Gravone and Prunelli) which implement less extensive farming practices (smaller estates, total fencing, limited use of summer pastures). Conversely, high and medium levels of IDWS were localized in the central higher regions of the island, where the pig farms are more extensive and isolated (Boziu, Verde, and Alesani). This association between higher levels of IDWS at municipalities with higher altitudes was significant (*p* < 0.05).

### Human Practices Driving IDWS

A number of quantitative indicators of interaction and potential management practices likely to influence those IDWSs were identified for farmers and hunters through exploratory data analysis. For the PCA analysis, only the two first dimensions were kept and the third dimension was also kept for the MFA. As a result, from the initial 34 variables, only the 16 categories that contribute the most to the two first dimensions were represented in Figure 4A (36). MFA highlighted a spatial proximity of all IDWSs indicators (Figure 4A), which appeared negatively correlated with practices such as total fencing of the farm perimeter or regular food supplementation. There was also a spatial proximity between practicing female sterilization and the observation of sexual interactions. On the third axis of the MFA (data not shown), the observation of wild boars appeared correlated to the use of summer pastures as well as the communal use of pastures between farmers.

Regarding hunters’ practices, the practice of selective shooting of feral pigs populations and cross bred animals that could have been seen on the farm (“Shoot feral pigs”), appears in proximity of the observed frequency of wild boars near the farm and the observation of agonistic IDWS (Figure 4B). The observation of sexual and agonistic IDWS is associated with a higher number of days per week dedicated to hunting activities (spatial proximity of these factors; Figure 4B). The type of hunting (driven hunts) seemed to equally influence negatively the observations of IDWS. Finally, being a SH showed to be negatively linked with IDWS reporting.

Results of the matrix identified correlations between some indicators of interaction and stakeholder’s management practices, which are listed in Table 3. There was a strong positive correlation between annual frequencies of agonistic and sexual interactions (*R* = 0.52).

**Table 3:** List of the most significant correlation coefficients between indicators of contact and farming or hunting practices (*p* < 0.05).

Practice	Interaction indicator	Correlation coefficient
**Farmers**		
Total fencing of farm perimeter (Y/N)	Number of observations of wild boar near farm	−0.69
Total fencing of farm perimeter (Y/N)	Annual number of interactions	−0.42
Additional food supply (Y/N)	Number of observations of wild boar near farm	−0.31
Sterilization of females (Y/N)	Annual number of observed fights	−0.29
Additional food supply (Y/N)	Observation of interactions (Y/N)	−0.26
Sterilization of females (Y/N)	Observation of interactions (Y/N)	0.26
Sterilization of females (Y/N)	Annual frequency of sexual interactions	0.25
More than 50 ha of outdoor area surface (Y/N)	Number of observations of wild boar near farm	0.28
Farming as main activity (Y/N)	Observation of interactions (Y/N)	0.31
Mating in non-fenced areas (Y/N)	Annual number of interactions	0.36
Communal use of pastures by different herds (Y/N)	Number of observations of wild boar near farm	0.37
Annual frequency of observed fights	Annual frequency of sexual interactions	0.52

**Hunters**		
Driven hunt (Y/N)	Annual frequency of sexual interactions	−0.33
Shooting feral pigs (Y/N)	Annual number of hybrid litters	0.32
Hunting practice (Y/N)	Number of observations of wild boar near farm	0.36
Hunting practice (Y/N)	Annual number of interactions	0.43

Regarding farming practices, the fact of having a farming estate totally fenced reduced the number of observations of wild boars around the farm (*R* = −0.69) and the number of observed IDWS (*R* = −0.42). Regular use of supplementary feeding was negatively correlated with the observation of wild or feral boars (*R* = −0.31) and non-specific IDWS (*R* = −0.26). The sterilization of females reduced the observation of agonistic IDWS (*R* = −0.29), but was positively correlated with the observation of non-specific IDWS (*R* = 0.25) and sexual interactions (*R* = 0.26). Farming surfaces larger than 50 ha or the communal use of pastures by different herds increased the observation of wild boars near the farm (*R* = 0.28 and 0.37, respectively). Finally, the non-confinement of reproductive sows was positively correlated with the annual frequency of non-specific IDWS (*R* = 0.36). For hunters, there was a strong correlation between the observation of non-specific IDWS and wild boars in proximity of pig farms (*R* = 0.4). In addition, we found an association between driven hunts and sexual interactions (*R* = −0.3).

## Discussion

IDWS are widespread worldwide and the number of shared pathogens between domestic and wild pig populations is considerable. As a result, there is a serious need to understand with more detail the nature and drivers of those interactions (37). In Corsica, IDWS are suspected to be intense and result in several pathogens such as bovine tuberculosis (3, 38), trichinellosis (4), or Hepatitis E virus (5, 39) circulating between both populations. Aujezsky disease (40, 41) remains present in Corsica despite being eradicated in the rest of the French territory, possibly as a result of IDWS (42). Others serious pig diseases such as African swine fever are endemic in neighboring Sardinia since 35 years partly as a result of IDWS (7, 8, 43). To the best of our knowledge, this work represents the most detailed study on IDWS in a specific area where they are suspected to be particularly intense and widespread. The use of the questionnaire method to collect traditional knowledge from rural stakeholders, which are privileged observers of natural events, proved to be efficient in capturing relevant ecological and epidemiological information from a large territory with limited time and resources. The preliminary identification of suitable seasons and locations for IDWS can allow to deploy more sophisticated and costly methods such as camera traps (12), or radio collars with data loggers (11) to monitor IDWS at a finer scale. As suspected, our study suggests that IDWS are common and widespread in the main outdoor pig production areas of Corsica, particularly in higher areas. Actually, the recorded levels of IDWS are probably underestimated for several reasons. First, we did not interview small-scale farmers keeping pigs outdoors, many of which are registered in the Animal Health Services records. This category of small-scale farmer (>300 individuals) is characterized by improvised farming facilities, poor reproductive management, and low biosecurity measures. These conditions should facilitate the incursions of wild boar, which are attracted by sows on heat or food remains (20, 44). Second, the observations collected referred to diurnal events and thus most nocturnal interactions, which might be equally common were unlikely to be observed by the respondents and not captured by the questionnaire.

In addition, some other sources of bias could have influenced the results of our study, which should be taken with caution until they can be validated with other studies or methodologies. Despite the sampling size was quite representative of the number of extensive farmers in each production area, the proportion of hunters interviewed was limited. Moreover, most of hunter’s observations refer mostly to the hunting season. In addition, a possible recall bias when asking for retrospective observations within a period of one whole year could also have influenced some of the responses. As a result, some data, particularly frequencies or durations of interactions, should be monitored with more sophisticated methods such as GPS collars or camera traps to confirm the stakeholder’s observations.

Nevertheless, the results obtained are biologically sound and the information collected by the different categories of stakeholders was consistently similar. For example, frequencies of sexual interactions, duration of intercourse, or season of sexual interactions were reported by the different stakeholders were very similar (Table 2). This suggests that the chosen method was efficient to collect abundant and detailed information, which is otherwise difficult to observe and quantify in a large territory.

Surprisingly, our questionnaire was equally able to capture some illegal or compromising practices such as inappropriate carcass management or the distribution of carcass offal to domestic pigs. This kind of behavior widespread is an extremely effective pathway for pathogen transmission between the domestic and wild compartments because it allows direct contact between individuals or their potentially infected tissues (45).

Indirect trophic interactions were less commonly observed but seemed to occur more regularly along the year, while direct sexually driven interactions (such as mating or fighting) appeared extremely common (Figure 2). As a measure of comparison, in a recent study in Switzerland, between 25 and 30% of the stakeholders had observed IDWS during the previous year to the interview and hybrid animals were reported in 5% of the 329 piggeries investigated (20). In our study, interactions were reported by 75% of the respondents and cross bred litters were reported (median 2 [1–4] per year) in 57% of the 65 farms investigated.

**Figure 5:**
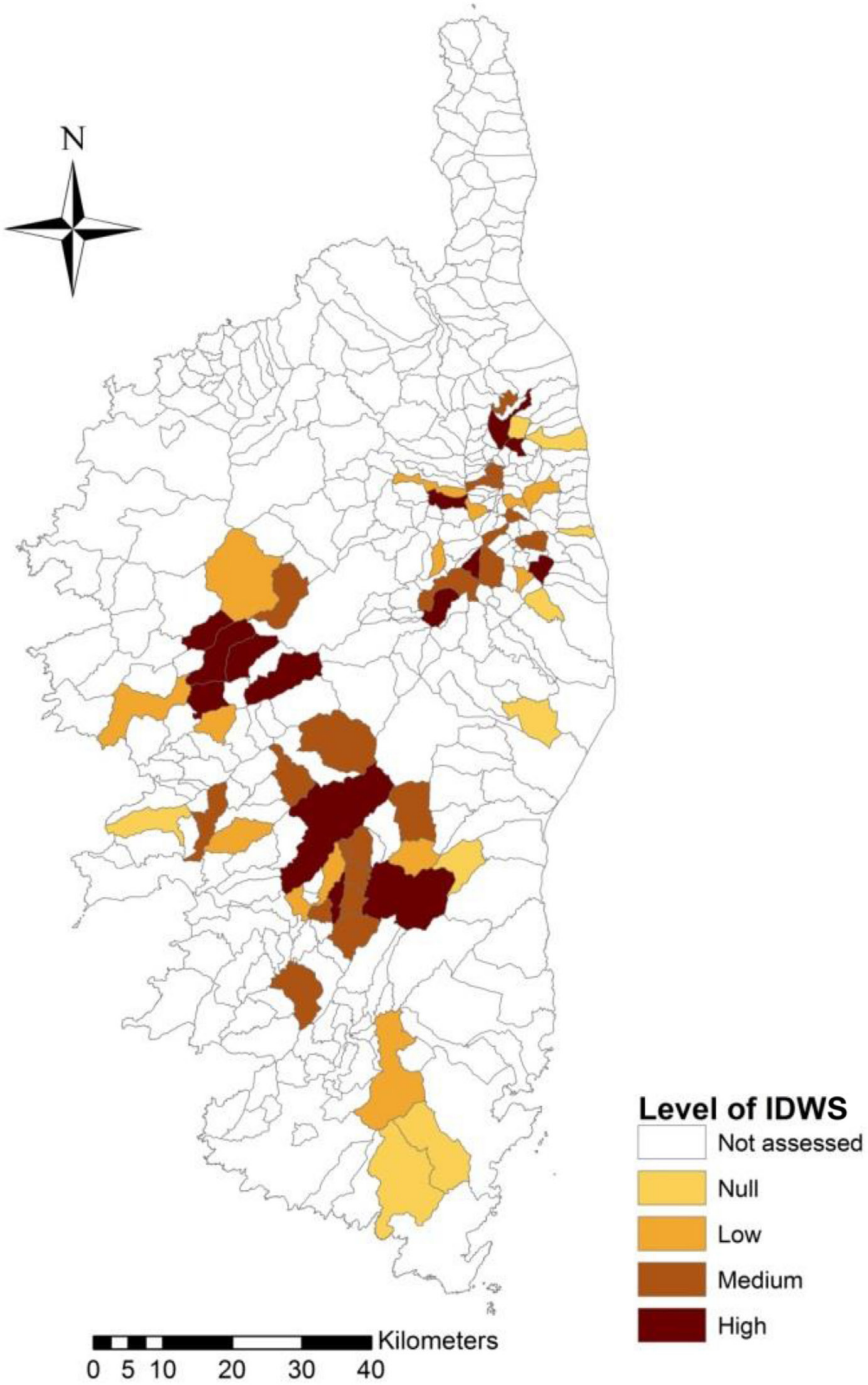
Map showing the average level of IDWS captured in the different interviews per municipality, classified as null, low, medium, or high.

The seasonal concentration of contacts in the autumn months (Figure 3) and at high altitude municipalities (Figure 5) suggests a specific risk of disease transmission in those periods and locations. Interestingly, it is worth noting that this seasonal pattern is consistent with observed seasonal outbreaks of African swine fever in wild boars as results of contacts with infected domestic pigs in Sardinia (8) and the Russian Federation (46). However, further research is necessary to confirm if this seasonal pattern is related to IDWS or to a potential observation bias, due to a higher level of human activity during summer months due to the hunting season.

A potential impact of reported sexual interactions is the possible increase of a hybrid pig population which roams free in the Corsican ecosystem interacting with domestic pigs and pure wild boar populations. Although insufficiently studied, these hybrids are suspected to have distinct reproductive, behavioral, and genetic patterns than pure wild boars that could make them exposed to higher disease burdens than those observed in pure wild boar populations (5, 47, 48). Considering the abundance and distribution of hybrid feral populations in Corsica, and the role they seem to play in similar settings (43), their potential epidemiological role in disease spread and maintenance deserves further investigation.

Many of the stakeholders identified during the questionnaires were implementing specific preventive mitigating measures to reduce IDWS. This could explain, for instance, some of the positive correlation presented in Table 3 between the number of hybrid litters and the shooting of feral pigs (*R* = 0.32), or the one between annual frequency of sexual IDWS and the sterilization of females (*R* = 0.25, Table 3). In some cases, these measures appear as having a non-negligible mitigating impact on the occurrence of IDWS, such as the case of driven hunts on sexual IDWS (*R* = −0.42), or the effect of supplementary feeding on the observation of non-specific IDWS (*R* = −0.26).

This suggests that there is some degree of risk perception among stakeholders and that the active promotion of some practices can potentially reduce the risk of disease transmission and spread. Furthermore, it highlights that local knowledge can be used to implement collective disease management strategies. Indeed, if this study increases knowledge about IDWS in extensive production system areas, it also reveals that farmers have an accurate knowledge on what happens with their animals, in and around their farm.

Our study provided useful information of the potential disease transmission risk of certain hunting and farming practices. This information should be used to engage a proactive communication and participative exchange of information with stakeholders in order to initiate decision-making processes or monitor risk mitigation strategies (49). From a management perspective, awareness campaigns should take advantage of the very seasonal (autumnal) and localized nature of many IDWS in areas of higher altitude, to concentrate efforts in those periods and areas (Figure 5). Similarly, farms with a high level of sexual interactions with wild boar could be more exposed to Aujeszky disease infection in domestic sows (42). In order to increase awareness, any communication on disease risks is likely to have a higher impact if it targets diseases of public health importance and emphasize interdependence between human, domestic animals, wildlife, and environmental health (50), such as trichinellosis (4), bovine tuberculosis (3), or HEV (39).

## Conclusion

The implementation of face-to-face semi-open questionnaires proved to be a useful tool to characterize IWDS in areas of extensive pig-farming production in Corsica, providing abundant and unique information on events that are otherwise difficult to observe. It also contributed to identify seasons and areas where IDWS are particularly common. This information can be used to concentrate efforts and resources in those particular spatial and temporal windows in order to implement risk-mitigating measures. In addition, IDWS can also be monitored and analyzed at a finer scale in order to provide data for the development of epidemiological models of disease transmission at the wildlife livestock interface (51, 52). Finally, this information can be used to engage participative decision-making processes on disease risk management with stakeholders.

## Author Contributions

FJ designed the study, analyzed some of the data, supervised field work, and wrote the paper; AR implemented interview, analyzed part of the data, and reviewed the manuscript; BT implemented interviews and analyzed some of the data; OM, FR, DC, DCh and FC supervised field work and contributed to design the sampling approach, EE analyzed part of the data, supervised the research project and contributed to the manuscript.

## Conflict of Interest Statement

The authors declare that the research was conducted in the absence of any commercial or financial relationships that could be construed as a potential conflict of interest.
